# Ultimate drivers of forced extra-pair copulations in birds lacking a penis: jackdaws as a case-study

**DOI:** 10.1098/rsos.231226

**Published:** 2024-03-27

**Authors:** Rebecca Hooper, Kathryn Maher, Karen Moore, Guillam McIvor, David Hosken, Alex Thornton

**Affiliations:** ^1^ Centre for Research in Animal Behaviour, University of Exeter, Exeter, UK; ^2^ NERC Environmental Omics Facility, School of Biosciences, The University of Sheffield, Sheffield, UK; ^3^ Faculty of Health and Life Sciences, University of Exeter, Exeter, UK; ^4^ Centre for Ecology and Conservation, University of Exeter, Penryn Campus, Penryn, UK

**Keywords:** forced, extra-pair, copulations, FEPC, jackdaw

## Abstract

Forced copulation is common, presumably because it can increase male reproductive success. Forced extra-pair copulation (FEPC) occurs in birds, even though most species lack a penis and are widely thought to require female cooperation for fertilization. How FEPC persists, despite a presumed lack of siring success and likely non-negligible costs to the male, is unknown. Using the jackdaw (*Corvus monedula*) as a case study, we use SNPs to quantify the extra-pair paternity rate through FEPC and evaluate explanations for the persistence of FEPC in species without a penis. We then collate evidence for FEPC across penis-lacking birds. Combining genetic and behavioural analyses, our study suggests that the most likely explanations for the maintenance of FEPC in jackdaws are that it provides a selective advantage to males or it is a relic. Our literature review shows that across birds lacking a penis, FEPC is taxonomically widespread, and yet, little is known about its evolution. A broader implementation of the approach used here, combining both genetic and behavioural data, may shed light on why this widespread sexual behaviour persists. Additional work is necessary to understand whether a penis is needed for paternity through forced copulation and to quantify the costs of FEPC.

## 1. Introduction

Copulations that are unsolicited and actively resisted by females (hereafter ‘forced copulations’) are common across the animal kingdom [1], occurring as obligate and facultative reproductive tactics [2,3]. Most commonly, forced copulation is a facultative, condition-dependent tactic where poor-quality males, unable to acquire mating through female choice, make the ‘best of a bad job’ [4–6]. For instance, smaller (lower quality) male camel crickets (*Pristoceuthophilus marmoratus*) force copulations with females more often than their larger conspecifics [7]. Similarly, in orangutans (*Pongo* spp.), females mate cooperatively with dominant males, but subordinate males often force copulations [8–10]. Forced copulation is evolutionarily favoured if it results in paternity and fitness gain [4,5,11], and it has been shown to enhance siring success in several species (e.g. [12–14]). For species with internal fertilization, it is widely thought that an intromittent organ (e.g. a penis) is necessary for forced copulation to result in siring success because, without a penetrative organ, female cooperation is required for successful insemination [15–17]. The occurrence of forced copulations in species without a penetrative organ is, therefore, puzzling. Here, we evaluate possible explanations for the existence of forced copulations in these species, focusing on birds.

Among birds, most species are socially monogamous [18], but forced extra-pair copulation (FEPC) is widespread [17,19–22]. FEPC is most commonly observed in the 3% of bird species that have a penis capable of penetrating the female cloaca [15,21,23,24], and FEPC is most often adopted as part of a mixed reproductive strategy (i.e. males have a monogamous partner and still engage in FEPC [21]). In these species, females may resist physically before, during and after the attempt [25]. Nevertheless, FEPC sometimes results in siring success, though success rates are typically low [26]. Thus, in birds with a penis, FEPCs are likely maintained as they increase male reproductive success [21], although direct evidence for this is rare (for detailed reviews of FEPC in birds with a penis, refer to refs. [21,23,27–29]). Here, we focus on the 97% of birds that lack a penis. In these species, the prevailing, although largely untested, viewpoint is that female cooperation (i.e. where the female positions herself so as to allow cloacal contact [21]) is needed for insemination and fertilization to occur [15–17]. Yet, for FEPC to persist, the fitness benefits for the male (e.g. siring success) must exceed costs. Male costs are difficult to quantify but are likely to be non-trivial. Males are at risk of injury during FEPC attempts owing to fights with the female or her partner [19,30]; energy must be expended to engage in FEPC [23]; and any paired males engaging in FEPC must leave their own partner unguarded. Leaving a partner unguarded may lead to egg damage or loss of paternity with social mates [30,31]. Stress and injury to the partner female during the male’s absence may also impact the success of the pair’s brood [30]. If FEPC cannot or rarely results in siring success, yet still persists in the population, this generates the question: what benefits exist to outweigh the costs of this behaviour?

There are seven different, non-mutually exclusive explanations for the evolution and maintenance of FEPC (electronic supplementary material, table S1). (i) FEPC is maintained by selection owing to direct fertilization enhancement. Here, fertilization success need only be great enough to outweigh the costs of engaging in FEPC. (ii) FEPC is an evolutionary ‘relic’. It is likely that ancestrally, all birds had penises [32]. The relic hypothesis suggests that while FEPC ancestrally provided paternity benefits, it now persists as a non-adaptive vestigial behaviour. (iii) FEPC is a pleiotropic byproduct of another trait that is selectively advantageous for males [6]. For example, high testosterone levels may be advantageous but result in hyper-sexual drive, leading to FEPC [28]. The remaining hypotheses posit that FEPC is selectively favoured, even though it does not directly result in paternity. These are (iv) the ‘Creation Of a Dangerous Environment’ (CODE) hypothesis [17] (but see §4 for a critique of this hypothesis), (v) the ‘territory signalling’ hypothesis (outlined in [30]), (vi) the ‘reproductive suppression’ hypothesis and (vii) the sperm turnover hypothesis. The CODE hypothesis posits that males use FEPC to create an environment of fear for females. This is beneficial for low-quality males because it selects for females to seek male protection (regardless of, or with a lower threshold for, quality) and to trade this protection for copulations. The ‘territory signalling’ hypothesis stemmed from observations that male jackdaws (*Corvus monedula*) produce loud copulation calls both when mating with social partners, and when engaging in FEPC. This could indicate that FEPC has a territorial function [30], whereby males attempt to claim future territory by forcing copulation with the resident female and signalling through loud copulation calls [30]. Gill *et al*. [30] also raised the possibility that FEPC may suppress the reproductive success of the victim female, for instance, through long-term negative effects of stress on her incubation or nestling–rearing behaviour. We label this the ‘reproductive suppression’ hypothesis, whereby males with a mated partner (paired males) use FEPC as a tool to suppress the reproductive success of their neighbours, thus decreasing competition for themselves and/or their offspring. Finally, FEPC could conceivably serve to enhance sperm quality. Baker & Bellis [33] suggested that masturbation evolved as a mechanism to maintain ejaculate quality through sperm turnover. Following this logic, males could be forcing copulation with non-partners to ensure only high-quality sperm fertilizes their social partner’s eggs. Indeed, in black-legged kittiwakes (*Rissa tridactyla*), hatching success and chick quality decrease significantly for pairs whose eggs are fertilized by older sperm [34], broadly suggesting that this could be feasible.

Here, we investigate FEPC within a single species, the jackdaw (*Corvus monedula*). Jackdaws are long-term socially monogamous, colonially breeding corvids [35–38]. Pairs have one reproductive attempt per year, and the fertile period of females is highly synchronous within populations [38]. Divorce is rare and pairs typically form in their first year and stay together for life [35,38]. Copulations tend to be visually concealed within nest cavities or nest boxes [30]—in over a decade of work at our field sites, we have never observed a copulation outside the nest. Neither field observations nor video footage recorded inside nest boxes have ever recorded cooperative extra-pair copulations in jackdaws (i.e. extra-pair copulation attempts with no active female resistance such as aggression or nest-box defence). However, in one population of jackdaws, 87.5% of monitored females experienced at least one FEPC attempt inside the nest box during a single breeding season [30]. Despite this, multiple studies, including one that found high rates of FEPC, report no or very low (<5%) rates of extra-pair paternity in the species [30,36,37,39]. Rates of extra-pair paternity can vary between populations though [40], highlighting the need for population-specific genetic pedigrees. Using a combination of pedigree analysis and behavioural data, we ask (i) what is the rate of extra-pair paternity through FEPC in our populations of jackdaws? And (ii) what is the most likely ultimate explanation(s) for why FEPC persists? To answer this question, we test predictions generated by the aforementioned hypotheses (see electronic supplementary material, table S1). Finally, we expand our taxonomic focus to investigate FEPC in all bird species lacking a penis. Although there have been several reviews of FEPC in the past [17,20–23,41], the most recent was over a decade ago, and numerous further studies have since reported FEPC behaviour in bird species that lack a penis. We therefore collated a list of species where FEPC has been reported, in order to (iii) evaluate the breadth of FEPC behaviour across birds lacking penises and to identify patterns of where FEPC occurs.

## 2. Methods

### 2.1. Ethics

This study was carried out with approval from the University of Exeter Research Ethics Committee (eCORN001858; eCORN002970), following the ASAB Guidelines for the Treatment of Animals in Behavioural Research and Teaching [42]. Subjects were not captured for the purpose of this study but had been previously captured and ringed by Cornish Jackdaw Project team members licensed by the British Trust for Ornithology and UK Home Office (project licence: 30/3261).

### 2.2. Study sites

From 2013 to 2019, the Cornish Jackdaw Project has collected behavioural and life history data from three sites in Cornwall, UK (Site X 50°17′32″N; 5°11′96″W; Site Y 50°11′26″N, 5°10′51″W; Site Z 50°11′56″N, 5°10′9″W). Across these field sites, ~85 nest-boxes were monitored throughout each breeding season, starting from when the jackdaws started building nests in March to when the chicks fledged in June. Nest-box owners were ringed after being caught through ladder trapping or using trapdoors at the nest-box [43]. Non-box-owning birds were also ladder-trapped and ringed for individual identification. Nest-box chicks were ringed at 25 days old. During ringing of chicks and adults, tarsus length (an indicator of structural size [44]), measured following ‘minimum tarsus’ guidelines [45], was recorded for each individual, and blood samples were collected by licenced members of the team and used for molecular sexing, as described by Griffiths *et al*. [46] and pedigree reconstruction.

### 2.3. Pedigree analysis

#### 2.3.1. Sampling, DNA extraction and sequencing

For chicks that survived until 25 days old, blood samples for DNA extraction were collected at ringing. In 2018 and 2019, nest-boxes with chicks <25 days old were monitored closely so that deceased chicks could be collected before removal from the nest by parents. Deceased chicks were frozen at −20℃ as soon after death as possible, and tissue samples from the liver were collected for DNA extraction. DNA was extracted from blood using Thermo Scientific GeneJET Whole Blood Genomic DNA Purification and from the liver using the QIAGEN DNeasy Blood & Tissue Kit, both following manufacturer’s protocols; see electronic supplementary materials for further details on DNA extraction and quality control.

Following DNA extraction, samples were selected based on fully sampled family units (i.e. broods where both social parents had been sampled), site (sample number was proportional to site size) and quality of DNA extraction. Five duplicate samples (from the same DNA extraction) were included in the sequencing pool in order to estimate the approximate sequencing error rate. We also included known social half-siblings (known through re-pairing of the social parent) so we could examine whether our final analysis had the power to detect half-sib relationships, which is key to estimating extra-pair paternity rates. A female with no known offspring or parents was also included, because she had been observed for several years to associate with a pair who were included (along with their offspring) in the sample selection. We also had video evidence of this female laying an egg in that pair’s nest-box (see §2.4).

Our final sample for sequencing consisted of 188 individuals (plus five duplicates) across two sites. 149 individuals were included from Sites Y and Z (treated as one site owing to gene flow; see §2.3.2), comprising 113 (plus two duplicates) offspring from 47 broods and 22 sibships. Two parent samples were also duplicates. From Site X, 43 individuals were included, which comprised 33 (plus one duplicate) offspring from 13 broods and 6 sibships. Quality control of DNA extracted from liver samples revealed a moderate level of degradation in many samples. Therefore, only six samples from deceased chicks were included in the analysis; all other samples were extracted from blood.

Samples were sent to Exeter Sequencing Services for library preparation and ddRAD sequencing; see electronic supplementary materials for detailed library prep and sequencing methods.

#### 2.3.2. Bioinformatic pipeline

Sequence data underwent strict quality filtering, presented in detail in the electronic supplementary materials. Following quality filtering, reads were run through process radtags in STACKS v.2.54 [47]. Reads were then aligned to the jackdaw reference genome (GenBank accession JABDSK000000000 [48]) using GSNAP/GMAP v.2020-10-27 [49] specifying a maximum of 10 mismatches (-m 10), an indel penalty (-i 2) and turning off terminal alignments (--min-coverage = 0.95). Only reads that aligned uniquely were retained (-n 1, --quiet-if-excessive) [50].

Following alignment, we retained samples with >1 million aligned reads. These were run through the ref_map module of STACKS, specifying a minor allele frequency of 0.15 (--min-maf 0.15), retaining one random SNP per read (--write-random-snp) and retaining only SNPs present in >50% of samples (-r 0.5). Finally, we used PLINK v.1.9 [51] to discard loci in linkage disequilibrium. To do this, we considered all individuals without sampled parents as ‘founders’ for Site Y + Site Z, two sites with substantial overlap and assumed gene flow (now referred to as Site YZ), and Site X, a separate site with little to no observed overlap with Site YZ. We tested for linkage disequilibrium using --indep, evaluating 50 SNP windows, 5 SNPs at a time with a variance inflation factor cut-off of 2 [52] and filtered out SNPs in linkage disequilibrium from our final datasets.

#### 2.3.3. Pedigree reconstruction

Pedigree reconstruction allows for both parental, sibship and half-sibship assignment. Thus, pedigree reconstruction allows us to understand whether chicks were the offspring of their social parents. We used Sequoia v.2.1.2 [53] in R v.4.0.2 [54] for pedigree reconstruction. Sequoia is an R package specifically designed for reconstructing multigenerational pedigrees using the SNP data. Unlike the traditional pedigree reconstruction software such as Colony [55], Sequoia does not require a candidate group of parents for each cohort, which would be difficult to accomplish with our data given multiple overlapping generations. Sequoia also explores a wider range of relationships than many other pedigree reconstruction software and runs quickly relative to alternatives [56].

Before pedigree reconstruction, SNPs and individuals were filtered to be scored for at least 30% of individuals or SNPs, respectively. Stricter filtering is typically recommended (Jisca Huisman, *personal communication*), but our SNP data were characterized by a high degree of missingness. Initial exploration showed that this level of filtering minimized the number of obvious assignment errors while maximizing the number of individuals included.

To reconstruct the pedigree, we first estimated the association between age difference and relationship type with the function MakeAgePrior(), using known and estimated birth years and the social pedigree. We then used the function *sequoia*(), specifying *Tassign* = 2 for conservative assignment of relatives, *Tfilter* = −4 to prevent filtering out of true relatives, *Complex* = ‘simp’ to not explicitly consider double relationships and *UseAge* = Extra. The genotyping error rate was set to 0.09, based on an estimation from four duplicated samples (see §3).

Following pedigree reconstruction and to further resolve relationships, we used CalcPairLL() with a flat age prior to explore relationship log likelihoods between all pairs of individuals (see electronic supplementary materials for an extended explanation). From CalcPairLL(), the Log Likelihood Ratio (LLR) of full-sibship versus unrelated (FS/U), half-sibship versus unrelated (HS/U) and parent–offspring versus unrelated (PO/U) was calculated. LLR(FS/U) and LLR(HS/U) for assigned full-siblings and assigned unrelated pairs were plotted against values for known social half-siblings (where a social parent is shared through a re-pairing event) to allow evaluation of the likelihood that individuals unassigned to their social sibship were the product of extra-pair paternity or maternity (half-siblings with their social sibship) or egg-dumping (unrelated to their social sibship; Jisca Huisman, personal communication; see electronic supplementary Materials).

### 2.4. Behavioural data

#### 2.4.1. Data extraction

We fitted cameras with microphones (380TVL CMOS camera, Handykam, Redruth, UK) inside nest-boxes during the 2014, 2015, 2018 and 2019 breeding seasons. Cameras were set to record starting from between 6.00 and 11.00 BST (*n* = 575 recordings). This captures the period when our previous observations, and other studies [30], indicate the majority of sexual behaviour occurs. In addition, a small subset was set to record in the afternoon (*n* = 8 recordings; 13.30–14:30 BST). Videos were recorded at three different stages of the breeding season: the nest-building stage, the incubation stage and the nestling stage, with one to three videos per pair per stage (total number of pairs = 130). The nest-building stage was filmed from when the building had begun, and a nest ‘cup’ was becoming apparent [57] until the nest was complete; the incubation stage was filmed 2–10 days after the female’s fertility window until hatching, and the nestling stage was filmed at 4–10 (nestling 1) and 17–23 (nestling 2) days following the first egg hatching. Note that videos were filmed for various research purposes, and in-line with these protocols, no videos were filmed during the female’s fertile period (5 days pre-clutch initiation to the penultimate lay date [30,36]).

Videos were coded by students following a training period. Using a standardized ethogram with the capacity for descriptive notes, video code was recorded using either the Microsoft Excel (from 2014 to 2015) or the Behavioral Observation Research Interactive Software, BORIS [58]. The collated coded data were searched for key terms that could potentially implicate an extra-pair intrusion event: ‘intr*’, ‘attack*’, ‘copul*’, ‘fight’, ‘aggress*’ and ‘defen*’. All videos that contained any of these key terms in their coded data were re-watched by RH, to verify whether an EPC was attempted. The confirmation of an EPC attempt was based on the identity of the intruding male, or if the individual was unidentifiable, on the pattern of behaviour observed relative to known EPC attempts (see electronic supplementary materials and Results). All EPC attempts (and associated male IDs) identified by R.H. were independently checked and confirmed by G.E.M.

For fine-sale behavioural analyses, all videos in the incubation stage that contained EPC attempts and a subset of incubation-stage videos that did not contain EPC attempts were re-coded by R.H. using a detailed behavioural ethogram (electronic supplementary material, table S4 ).

#### 2.4.2. Statistical analysis

We used GLMMs to investigate differences in fine-scale behaviours between within-pair and extra-pair copulation attempts, while accounting for potential effects of individual characteristics (age and skeletal [tarsus] size) and temporal variation (across years and time of day; see tables 1 and 2 and electronic supplementary materials: Covariate Inclusion). Differences between behaviours during within-pair and extra-pair copulation attempts allowed us to identify whether EPCs were forced. Following the identification of FEPC attempts, we then identified fine-scale behavioural patterns of jackdaws in relation to FEPC, in order to test predictions of the hypotheses presented in electronic supplementary material, table S1. Statistical analyses were conducted using glmmTMB v.1.0.1 [59] in R v.4.0.2 [54]. For fine-scale behavioural models, the response variable was the number of seconds for which the behaviour occurred. For models comparing copulation length and copulation visit length between FEPC attempts and within-pair copulation attempts, no offset was necessary; for all other behavioural models, an offset of either video duration (seconds) was used or, for models exploring time males spent with the females, the sum of seconds the female spent in the nest-box. The most appropriate error structure was selected based on model diagnostic plots (using DHARMa) and the lowest AIC. Nbinom2 (negative binomial with a quadratic parameterization) was selected for all models in tables 1 and 2 (behavioural models), except table 2 model 2 which used nbinom1 (negative binomial with a linear parameterization). For non-behavioural correlates of FEPC attempts, logistic regressions with a binomial error structure and a probit link function were used. Pair ID was included as a random effect in all models, except for models comparing attempted within-pair copulation and attempted FEPC copulation length and copulation visit length, where male ID was the random effect. Models were validated by testing for normality of residuals, overdispersion and zero inflation using DHARMa [60]. Where zero inflation occurred, a zero inflation term was included in the model (table 1, model 2; table 2, model 1). Influential points were identified as those that were more than four times the mean Cook’s Distance [61]. Where full models and those with influential points removed are qualitatively identical, the latter are presented. Where a key result differs between models (*n* = 3 models: table 2, models 1, 2 and 3), both are reported in the main text while the model without influential points is reported in the associated table, with additional detail in the electronic supplementary materials. The model comparing attempted within-pair copulation and attempted FEPC visit lengths did not perform well with the inclusion of extreme values (*n* = 4, see electronic supplementary materials); so, these were removed for this model (results remained qualitatively identical). The model investigating the relationship between the female structural size and FEPC attempts did not perform well with influential points excluded so the full model is presented, again with further detail in the electronic supplementary materials. Note that the sample size (*n* videos and *n* pairs) per model varies because (i) some information (such as the tarsus size) is missing for some individuals, and covariates per model vary (tables 1–2; electronic supplementary materials) and (ii) a varying number of influential points (min = 0 pairs, max = 5 pairs) were removed per model.

**Table 1.** Resident male behaviour and FEPC. The response variable was number of seconds engaged in the behaviour, with an offset of video length for model 1, and female time in the nest-box in model 2. Pair ID was included as a random effect in both models, and both used an nbinom2 error structure. A zero inflation term was included in model 2. Both models have influential points removed; key results (FEPC in video; days since female fertile) were qualitatively identical with their inclusion. Bold indicates significant results.

model n	response	n videos	n pairs	fixed effects	*β*	s.e.	Χ^2^	95% CI (lower)	95% CI (upper)	*p*‐value
1	proportion of video resident male is vigilant	73	41	**FEPC in video (yes)**	**−1.109**	**0.498**	**4.951**	**−2.085**	**−0.132**	**0.026**
				**video starting time**	**−0.407**	**0.098**	**17.141**	**−0.599**	**−0.214**	**0.00003**
				**male tarsus**	**−0.231**	**0.112**	**4.226**	**−0.451**	**−0.011**	**0.040**
				**female tarsus**	**0.277**	**0.100**	**7.643**	**0.081**	**0.473**	**0.006**
				**male’s minimum age**	**0.324**	**0.109**	**8.866**	**0.111**	**0.537**	**0.003**
				**year (15)**	**−0.319**	**0.844**	**11.953**	**−1.973**	**1.335**	**0.008**
				**year (18)**	**0.311**	**0.913**		**−1.478**	**2.100**	
				**year (19)**	**−1.148**	**1.011**		**−3.131**	**0.834**	
				days since female fertile	−0.052	0.091	0.334	−0.230	0.125	0.563
2	proportion of video resident male is with with female	69	40	**FEPC in video (yes)**	**−0.918**	**0.309**	**8.811**	**−1.525**	**−0.312**	**0.003**
				**video starting time**	**−0.116**	**0.057**	**4.065**	**−0.228**	**−0.003**	**0.044**
				**male tarsus**	**−0.252**	**0.070**	**12.939**	**−0.389**	**−0.115**	**0.0003**
				**female tarsus**	**0.220**	**0.069**	**10.129**	**0.085**	**0.356**	**0.001**
				**male’s minimum age**	**0.181**	**0.064**	**8.036**	**0.056**	**0.306**	**0.005**
				**year (15)**	**0.842**	**0.536**	**14.596**	**−0.209**	**1.893**	**0.002**
				**year (18)**	**1.065**	**0.556**		**−0.026**	**2.155**	
				**year (19)**	**0.094**	**0.617**		**−1.117**	**1.304**	
				days since female fertile	−0.052	0.057	0.829	−0.163	0.060	0.363

**Table 2.** Resident female behaviour and FEPC. The response variable was number of seconds engaged in the behaviour, with an offset of video length. Pair ID was included as a random effect in models 1 and 3, while video ID was used as a random effect in model 2. Models 1 and 3 used an nbinom2 error structure; model 2 used an nbinom1 error structure. A zero inflation term was included in model 1. All models have influential points removed. Key results differed with their inclusion; see §3 and electronic supplementary materials. Bold indicates significant results.

model n	response	n videos	n pairs	fixed effects	*β*	s.e.	Χ^2^	95% CI (lower)	95% CI (upper)	*p*‐value
1	proportion of video resident female is vigilant	73	41	**FEPC in video (yes**)	**0.732**	**0.365**	**4.028**	**0.017**	**1.448**	**0.045**
				video starting time	0.028	0.075	0.144	−0.118	0.175	0.704
				**female tarsus**	**0.212**	**0.092**	**5.308**	**0.032**	**0.393**	**0.021**
				male tarsus	−0.071	0.101	0.493	−0.268	0.127	0.482
				female’s minimum age	−0.048	0.098	0.235	−0.240	0.145	0.628
				year (15)	0.606	0.928	2.339	−1.213	2.424	0.505
				year (18)	0.106	0.984		−1.823	2.035	
				year (19)	0.125	1.065		−1.963	2.213	
				days since female fertile	0.036	0.068	0.277	−0.097	0.168	0.598
2	proportion of time resident female who experienced FEPC is vigilant	24	12	**pre/post-FEPC (post**)	**−0.609**	**0.250**	**5.937**	**−0.119**	**−0.609**	**0.015**
				**video starting time**	**1.971**	**0.746**	**6.986**	**3.433**	**1.971**	**0.008**
				**female’s minimum age**	**1.022**	**0.501**	**6.930**	**3.656**	**2.036**	**0.041**
				**year (15**)	**2.036**	**0.827**	**1.254**	**2.394**	**0.027**	**0.031**
				**year (18**)	**0.027**	**1.208**		**0.140**	**−0.187**	
				days since female fertile	−0.187	0.167	**4.157**	2.005	1.022	0.263
3	proportion of video resident female is incubating	69	41	**FEPC in video (yes**)	**−0.073**	**0.034**	**4.612**	**−0.139**	**−0.006**	**0.032**
				video starting time	−0.007	0.007	0.920	−0.020	0.007	0.338
				female tarsus	−0.002	0.006	0.135	−0.015	0.010	0.713
				**male tarsus**	**−0.019**	**0.007**	**7.378**	**−0.033**	**−0.005**	**0.007**
				female’s minimum age	-0.004	0.008	0.211	−0.019	0.012	0.646
				**year (15**)	**−0.149**	**0.080**	**8.530**	**−0.306**	**0.009**	**0.036**
				**year (18**)	**−0.083**	**0.085**		**−0.250**	**0.083**	
				**year (19**)	**−0.067**	**0.090**		**−0.244**	**0.110**	
				male-to-female food-sharing	−0.001	0.005	0.044	−0.011	0.009	0.833
				days since female fertile	−0.005	0.006	0.555	−0.017	0.008	0.456

### 2.5. FEPCs beyond jackdaws

To understand the prevalence of FEPC in birds lacking penises, we collated species reported to engage in FEPC from previous reviews [17,19–21] and used Google Scholar to search for additional reports of FEPC, using the terms ‘bird’ + ‘forced copulation’ or ‘rape’ or ‘FEPC’ or ‘force’ + ‘copulation’. We excluded species with penises and included only species where active female resistance to attempted male copulation has been reported. For example, while one forced extra-pair copulation was suspected to have occurred in the tree swallow (*Tachyncineta bicolour*) when an extra-pair male was observed chasing a female into her nestbox [62], active female resistance was not reported and so, to be conservative, this species was not included. In addition, if species were included in review papers as species in which FEPC occurs, but the primary literature did not describe female resistance to male copulation attempts, these species were excluded. If the primary source describing FEPC could not be accessed (e.g. in cases of personal communication between review authors and researchers), these species were included. Given that extra-pair behaviour can differ drastically between captive and wild populations (e.g. [63]), we only included species where FEPC has been recorded in the wild. In order to identify broad-scale patterns in FEPC occurrence, we also collected information on species’ mating system, whether they are colonial breeders, the identity of the male engaging in FEPC (e.g. if he was known to be a neighbour), and whether unforced EPC has also been observed.

## 3. Results

### 3.1. Pedigree analysis

#### 3.1.1. Bioinformatic pipeline

Following SNP discovery and filtering out of SNPs in linkage disequilibrium, 901 SNPs were retained for Site YZ and 770 SNPs were retained for Site X; see electronic supplementary materials for additional information regarding sequencing output and quality control.

#### 3.1.2. Pedigree reconstruction

*Site YZ:* 901 SNPs and 117 individuals were loaded into Sequoia. Following 30% missingness filters, 757 SNPs and 93 individuals were retained (17 parents, 76 offspring, 44 broods and 19 sibships). Pedigree reconstruction with *sequoia*() and inspection of pairwise relationships (calculated from *CalcPairLL*(); see electronic supplementary materials), both of which were blind to the social pedigree, found that 67 of the 76 offspring clustered into their known social sibships and were the offspring of their social parent(s), where their social parent(s) was retained in the analysis.

The nine offspring who did not have convincing support for full sibship with their social sibships were investigated in detail, and a full discussion of each case is included in the spection of pairwise relationships electronic supplementary materials. In brief, there were two likely cases of egg-dumping in two different nests. Egg-dumping is where non-resident females lay eggs in a nest-box they do not own, and the resident male is not the father. This is one of the first reported instances of such behaviour in jackdaws, though there is some tentative evidence from the Israeli population [39]. We found two ambiguous cases of egg-dumping or half-sibship within a social sibship (i.e. we could discern only that these individuals were not full siblings with the rest of the clutch), and five likely cases of half-sibship within a social sibship. Half-sibship within a social sibship can be the result of either extra-pair maternity, where non-resident females lay an egg sired by the resident male in his nest-box or extra-pair paternity, where resident females lay an egg sired by a non-resident male in the nest-box. Of the five likely cases of half-sibship, two are highly likely to be the result of extra-pair maternity through a ‘follower’ relationship, where an extra-pair female was often observed with a nest-box-owning pair and was recorded on video laying an egg in their nest-box. Combining SNP and behavioural data, this extra-pair female is likely to be the mother of two individuals from this nest-box across two different years (2017 and 2019), with the resident male likely to be the father. The genetic evidence suggests that this male may also have sired another chick in this nest-box whose mother was neither the resident female nor the known follower female. This indicates that the resident male may have sired offspring with a total of three females, all while maintaining his pair-bond with one partner. This is the first reported instance of extra-pair maternity in jackdaws.

A further case of likely half-sibship was identified to most likely be the result of extra-pair paternity, although we could not rule out extra-pair maternity, while the remaining case was unresolved (i.e. either extra-pair maternity or paternity). Of the two ambiguous cases of egg-dumping or half sibship, one was most likely the result of either egg-dumping or extra-pair maternity, while the final case was unresolved (i.e. could be the result of egg-dumping, extra-pair maternity or extra-pair paternity).

Fertilization as a result of EPC would most likely be reflected as extra-pair paternity within a social sibship. Extra-pair maternity as a result of fertilization through EPC is considered highly unlikely, given that following an EPC event, females would need to (i) know the nest location and (ii) take the risk of entering the nest-box of the male who engaged in extra-pair copulation with her and fertilized her egg, and it is not immediately obvious why this would occur. Furthermore, across 8 yr of field observations, we have never observed a nest-box-owning, breeding female enter another pair’s nest-box around the date of laying. In contrast, we have observed a follower female laying her and the resident male’s eggs inside his nest-box, so we therefore suggest that cases of extra-pair maternity in this population are most likely owing to unobserved cases of follower females. Indeed, while we present the first evidence that jackdaw males and follower females produce offspring together, follower relationships are known to occur in other jackdaw populations [37,38], indicating that it may not be an extremely rare behaviour. To calculate the potential rate of fertilization through EPC, we, therefore, considered only extra-pair paternity. Including unlikely but potential cases of extra-pair paternity, this population has a potential rate of fertilization through EPC of 0/76–3/76 (0–3.95%).

*Site X:* 770 SNPs and 38 individuals were loaded in Sequoia. Following 30% filters, we retained all SNPs and 37 individuals (9 parents, 28 offspring, 13 broods, 6 sibships). We identified no cases of egg-dumping and one potential case of half-sibship within a social sibship, which was most likely the result of extra-pair maternity, although could potentially be a case of extra-pair paternity instead (see electronic supplementary materials). Thus, the potential rate of extra-pair paternity through EPC is considered to be 0/28–1/28 (0–3.57%).

Across both sites, the maximum rate of fertilization through EPC, including unlikely but potential cases, is 4/104 (3.85%).

### 3.2. Behavioural data analysis

Of 1786 h of nest-box video filmed and coded across 575 videos and 130 pairs, 87 within-pair copulations across 39 pairs (1–4 per pair) were observed. Of these, 35 (40%) were in the nest-building (pre-fertile) stage (301.84 video hours), 52 (60%) occurred during the incubation (post-fertile) stage (708.51 video hours), and none occurred in the nestling-rearing (post-fertile) stage (776.01 video hours). In total, we recorded 17 attempted EPCs (*n* = 5 events at Site X, *n* = 12 events at Site YZ). Of these, one attempted EPC was in the nest-building stage and 16 were in the incubation stage, observed across 14 videos (two videos contained two attempted EPCs). No videos in the nestling–rearing stage contained EPC or attempted EPC.

For the fine-scale behavioural analysis of videos in the incubation stage, all videos containing attempted EPC (*n* = 14 videos) and 87 randomly selected videos were re-coded by the lead author using the fine-scale behavioural ethogram (electronic supplementary material, table S4 ), equating to 290.28 h of recording and 63 jackdaw pairs.

#### 3.3. Intruding male ID

In 13 of the 17 attempted EPCs (where extra-pair copulation was observed), we could establish with certainty that the intruding male was not the resident male (see electronic supplementary materials). In six attempted EPCs, the intruder was identifiable owing to a full set of visible leg rings, and in one case, the identity was ambiguous (two possible identifications) owing to one unclear ring. In five of the six cases of confident identification, the intruder was a paired adult male who owned a neighbouring nest-box and who had a partner either in the process of laying or incubating eggs (in two of these instances, the intruder was the same male forcing copulation with different neighbouring females). In the sixth case of confident identification, the male was known to be an adult but we had no further information about him. In the case of ambiguous identification, the male was either a paired adult neighbour or an adult male for whom we had no further information. Four of the five males who we could confidently identify had bred in nest-boxes the year preceding the attempted EPC. Two of these males had failed to fledge any offspring despite eggs being laid, one had built a nest with his partner but she did not lay eggs, and the final fledged two offspring (for comparison, the average reproductive success in that year was 1.85 ± 0.88 chicks per pair). Based on behavioural differences between within-pair copulation and definite cases of attempted EPC (see below), the four putative cases of attempted EPC, which entirely matched the behavioural profile of known attempted EPCs, are considered highly likely to be true cases of attempted EPC and are henceforth treated as such.

Note that there was no overlap between the males identified as potential extra-pair sires and those observed engaging in FEPC.

### 3.4. Are EPCs forced?

Attempted EPCs (100%) co-occurred with female nest-box defence (charging towards the nest-box entrance, as described in [30] and/or prolonged female aggression (pecking/kicking the head and body of the intruder or turning around and fighting with claws) that occurred throughout the copulation attempt (electronic supplementary materials, video S1). We did not observe any overt instance of female copulation solicitation (bending the tail upward and to the side, often accompanied by horizontal tail shaking [30]; although we cannot conclusively rule out the occurrence of subtle solicitation behaviours). No attempted EPCs appeared to be completed by the intruder (wing flapping of less than 2 s indicated an aborted attempt [30]), and none were associated with affiliative behaviours (allopreening, contact sitting/standing; see electronic supplementary material, table S4). In two cases, the resident male returned during the attempted EPC; in one case, the intruder immediately left the nest-box, and in the other, a fight ensued between the resident male and the intruder for ~22 s until the intruder left (electronic supplementary materials, video S2).

**Figure 1.**
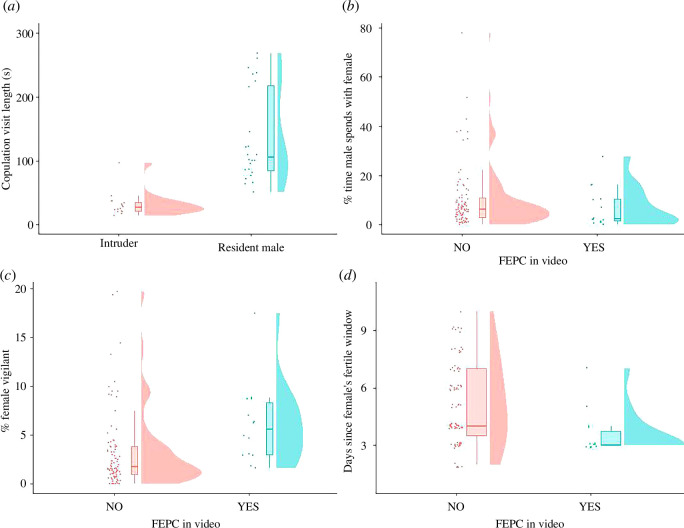
(*a*) Length of visits (in seconds) in which copulation occurred for intruders (FEPC attempts) and resident males (within-pair copulation attempts). Intruder visit length was significantly shorter than resident male visit length (*n* = 35 males, *p* < 0.0001). Note that copulation visits of >300 s (nvisits = 5, nmales = 4, all of whom were resident males) have been removed for visualization purposes; these values were 466, 615, 715, 1143 and 2338 s. (*b*) Percentage of time the resident male spends with the female, out of total female time in the nest-box, for videos with and without FEPC. Males spent significantly less time with their partners in videos where FEPC attempts occurred. Females whose partners spent more time in the nest-box were significantly less likely to experience FEPC attempts (*n* = 410 pairs, *p* = 0.003). (*c*) Percentage of video in which the female engages in vigilance across videos with and without FEPC attempts. Females engaged in significantly more vigilance in videos with FEPC attempts (*n* = 41 females, *p* = 0.045). (*d*) Days since the female’s fertile window and whether an FEPC attempt occurs. The closer to the fertile period the female was, the more likely she was to experience an FEPC attempt (*n* = 41 females, *p* = 0.03). All figures show raw data with influential points included (see §2). Circles indicate raw data-points; boxplots show the median, lower and upper quartile of the data distributions; violin plots show the density curves of the data distributions.

The fine-scale behavioural dataset included 24 videos containing 32 within-pair copulations (across 29 male visits). In contrast to attempted EPC, no within-pair copulations or attempted copulations were preceded by nest-box defence by the female, and 100% of within-pair copulations or attempted copulations were associated with male affiliation towards the female prior to copulation. However, no copulations were solicited by the female (using behavioural criteria outlined in [30]). Only 28% of within-pair copulations appeared to be completed, and 47% were associated with female aggression (turning to her partner with an open beak or pecking at her partner’s beak/head). In contrast to attempted EPCs, female aggression during within-pair copulations was brief and caused the male to cease his copulation attempt. While the length of copulation was not significantly different between within-pair copulations and attempted EPCs (*β* = −1.38, SE = 2.51, χ^2^ = 0.30, 95% CI [−6.29,3.54], *p* = 0.58), the length of the visit in which the copulation occurred was significantly longer in within-pair copulations than attempted EPCs (*β* = 1.50, SE = 0.20, χ^2^ = 60.25, 95% CI [1.12,1.87], *p* = <0.0001; figure 1*a*); see electronic supplementary materials, video S3 for an example of within-pair copulation.

Considering the female’s nest-box defence, prolonged attack behaviours and the lack of the intruder’s pre-copulatory routines (specifically affiliation towards the female), it appears that all identified EPC attempts were indeed forced, and so will, henceforth, be referred to as FEPC.

### 3.5. Resident male behaviour and FEPC

All FEPC attempts occurred when the resident male was absent from the nest-box. As presented in table 1, Model 1, males whose partners experienced FEPC attempts (FEPC in video: Yes) spent a lower proportion of time in vigilance (‘peeking’, see electronic supplementary material, table S4) at the nest-box (*β* = −1.11, SE = 0.50, χ^2^ = 4.95, 95% CI [−2.09,−0.13], *p* = 0.03) in the period preceding the FEPC attempt, relative to males whose partners did not experience FEPC attempts.

As presented in table 1, Model 2, males whose partners experienced FEPC attempts, spent a lower proportion of time with them at the nest-box in the period preceding the FEPC attempts (measured as time the pair spent together controlled for overall time the female spent at the nest-box; *β* = −0.92, SE = 0.31, χ^2^ = 8.81, 95% CI [−1.53,−0.31], *p* = 0.003), relative to males whose partners did not experience FEPC attempts.

Patterns of male vigilance and time together at the nest-box also varied with video starting time, male tarsus size, female tarsus size, male age and year.

### 3.6. Resident female behaviour and FEPC

As presented in table 2, Model 1, with influential points removed females spent significantly more time in vigilance when FEPC attempts occurred relative to when they did not (*β* = 0.73, SE = 0.36, χ^2^ = 4.03, 95% CI [0.02,1.45], *p* = 0.045, figure 1*c*). The female tarsus size also predicted the amount of time females spent in vigilance.

As presented in table 2, Model 2, females who experienced FEPC spent significantly more time in vigilance before the FEPC attempt than after it (*β* = −0.61, SE = 0.25, χ^2^ = 6.99, 95% CI [−1.10,−0.12], *p* = 0.01), suggesting that external cues alerted them to the possibility of danger. The proportion of time spent in vigilance for females who experienced FEPC was also influenced by video starting time, female age and year.

As presented in table 2 , Model 3, females spent significantly less time incubating their eggs in videos with FEPC attempts (*β* = −0.07, SE = 0.03, χ^2^ = 4.61, 95% CI [−0.14,−0.01], *p* = 0.03). The resident male tarsus size and year also influenced time females spent incubating. Without the removal of influential points (*n* = 4, *n* = 1 and *n* = 4 pairs for each model, respectively; see electronic supplementary material for further details), there was no significant difference in either the vigilance model (no FEPC attempt versus FEPC attempt: *β* = 0.49, SE = 0.37, χ^2^ = 1.74, 95% CI [−0.24,1.21], *p* = 0.19; pre-FEPC attempt versus post-FEPC attempt: *β* = −0.35, SE = 0.28, χ^2^ = 1.52, 95% CI [−0.90,0.20], *p* = 0.22) or incubation duration in videos with/without FEPC attempts (*β* = −0.01, SE = 0.04, χ^2^ = 0.02, 95% CI [−0.09,0.07], *p* = 0.87).

### 3.7. Predictors of FEPC

The structural size of the female and her partner were unrelated to the female’s risk of experiencing an FEPC attempt (female tarsus size: *β* = −0.18, SE = 0.12, χ^2^ = 2.09, 95% CI [−0.15, 0.06], *p* = 0.15; male tarsus size: *β* = −0.05, SE = 0.21, χ^2^ = 0.05, 95% CI [−0.45, 0.36], *p* = 0.83). However, the closer to her fertile window the female was, the more likely she was to experience an FEPC attempt (*β* = −0.45, SE = 0.21, χ^2^ = 4.56, 95% CI [−0.53, −0.04], *p* = 0.03, figure 1*d*). It is possible that the greater risk of FEPC closer to the fertile window could result from changes in resident male behaviour; for instance, if males left their partner alone for longer periods. However, we found no evidence that males changed either vigilance (*β* = −0.05, SE = 0.09, χ^2^ = 0.33, 95% CI [−0.23, 0.13], *p* = 0.56; table 1, model 1) or time with the female (*β* = −0.05, SE = 0.06, χ^2^ = 0.83, 95% CI [−0.16, 0.06], *p* = 0.36; table 1, model 2) relative to number of days post-fertile.

### 3.8. FEPCs in other birds

We identified 48 bird species lacking penises where FEPC has been reported, nine of which were not included in previous reviews (see electronic supplementary material Data). These species spanned 23 families and 11 orders, out of a total of 22 bird orders that lack penises. Laridae (gulls), Ardeidae (herons) and Corvidae (corvids) had the largest number of species recorded to engage in FEPC (*n* = 9, 7, 7, respectively). Of all included species, 98% of those with known mating systems (*n* species = 47) are always or primarily socially monogamous (*n* species = 46), while 75% (*n* species = 36) are always or sometimes colonial breeders. In 20 of the 48 species, the male was recorded in at least one instance to be a neighbour of the target female. In 19 of the 48 species, the male was recorded in at least one instance to be a paired individual (i.e. to have a mating partner), while in four species, the male was known in at least one instance to be unpaired. Just under half of the species recorded (48%, species = 23) are reported to engage in unforced EPC (i.e. where the female does not resist or actively seeks EPC with non-partner males) as well as FEPC.

No studies have directly investigated whether FEPC can lead to fertilization in species lacking penises, but there is evidence to suggest FEPC can lead to fertilization in the stitchbird (or hihi; *Notiomystis cincta*) [12,64]. In addition, FEPC may lead to fertilization in American crows (*Corvus brachyrhynchos*) [65], where in one study, population 19% of offspring were sired by extra-pair males, likely but not certainly through FEPC. In contrast, the limited evidence available indicates that FEPC rarely or never results in fertilization in jackdaws (here, [30]), western gulls (*Larus occidentalis*) [66] and black-legged kittiwakes (*Rissa tridactyla*) [67].

## 4. Discussion

If bird species lacking a penis rarely or never achieve fertilization through FEPC, then the reason forced copulation (an apparently costly behaviour) occurs is puzzling. Generally, we do not know whether FEPC leads to siring success, but in jackdaws, it seems that it rarely, if ever, does. Hence, explanations unrelated to siring success may be needed to explain why FEPC occurs in this species. Here, we discuss our findings in jackdaws, explore the occurrence of FEPC across penis-lacking birds, identify knowledge gaps and suggest future research directions.

Despite multiple detailed observational studies of various wild jackdaw populations (e.g. here, [30,36,37]), including through the use of video cameras within the nest-box (where copulations virtually always occur in jackdaws) [30], females have never been seen to engage in cooperative EPC. Thus, the rate of extra-pair paternity is very likely to reflect the rate of paternity through FEPC in jackdaws. We found that both our populations had an FEPC paternity rate of 0–4%. Paternity studies of multiple wild jackdaw populations have revealed similarly low levels of extra-pair paternity [30,36,37,39], and this is true even in populations where 87.5% of monitored females experienced at least one FEPC attempt in a single year [30]. While most previous studies did not observe behaviours within the nest-box, multiple authors also report behaviours in-line with FEPC (i.e. non-partner males entering a nest-box with a lone female inside: Chen, R., personal communication [36]). Unfortunately, owing to our sampling protocol, it was not possible to estimate the rate of FEPC attempts (i.e. how many FEPC events each female in a population experiences per breeding season) in our study system. Nevertheless, given the extremely high rate of FEPC in another population [30], and low levels of extra-pair paternity reported across all jackdaw paternity studies [30,36,37,39], current evidence indicates that while FEPC attempts are common in jackdaws, they rarely, if ever, result in paternity.

If FEPC never or very rarely increases siring success, why do males engage in this behaviour? This is especially perplexing considering the potential costs of FEPC. While costs are inherently difficult to quantify in a wild and long-lived species, FEPC costs to males appear to be non-trivial in jackdaws. Specifically, both we and Gill et al. [30] observed fights between females and the male attempting FEPC. Moreover, if the partner male returned during the FEPC attempt, escalated fighting sometimes occurred. Such conflict is likely to result in injury given that pecking and kicking occurred for prolonged periods in a confined space. Indeed, in a Dutch population of jackdaws, an individual was killed after intruding into the nest-box of another pair (Ronald van Harxen*,* personal communication). Attempting FEPCs must also involve energy expenditure. In addition to these apparent costs of fighting, because males attempting FEPC only ever entered a nest-box when the resident male was absent, they must have invested time monitoring the victim’s nest-box (also refer to [30]). Given that all but one of the identifiable males attempting FEPC were breeding neighbours whose partner was laying or incubating eggs, these males also traded time foraging for themselves and their partner (males food-share with females while they incubate: [38]) with time attempting FEPC. Moreover, when seeking FEPC males left their mate alone at the nest-box, leaving their partner vulnerable to FEPC attempts (and the associated injury, stress and energy expenditure) and the pairs’ eggs (if laid) vulnerable to damage [30]. Taken together, it seems likely that males experience the costs of attempting FEPC.

Of the proposed hypotheses explaining why FEPC occurs, two seem most consistent with our findings: the immediate fertilization enhancement hypothesis and the relic hypothesis (electronic supplementary material, table S1). The immediate fertilization enhancement hypothesis is only supported if the rate of fertilization via FEPC is non-zero. Given that the estimated rate is 0–4%, we cannot rule this explanation out, and FEPC may be maintained by a small but significant selective advantage it potentially offers males. However, if true, how are males able to successfully transfer sperm and gain paternity without female cooperation? This is unclear and requires further research. The relic hypothesis, on the other hand, posits that FEPC originally benefitted males through increased paternity and persists as a vestigial behaviour even though it is no longer advantageous. All predictions of the relic hypothesis were met (but see the caveats on costs below). Specifically, we found that siring success through FEPC is rare or non-existent and that FEPC is more likely to occur closer to the female’s fertile period, independent of the resident male’s behaviour. This suggests that intruders try to access females when they are fertile, implying that the behaviour originally evolved for paternity enhancement. Indeed, resident males increase their mate-guarding efforts when their partner is fertile [30], which suggests that cuckoldry avoidance has evolved in this species [68]. Furthermore, three of the four identifiable males for whom we had breeding information in the year previous to their FEPC attempt had failed breeding attempts in that year. This suggests that males may adopt a ‘best of a bad job’ strategy, where they try to increase reproductive success by pursuing fertilization outside of the pair-bond. Such strategies are commonly observed in species where forced copulation leads to fertilization [2]. While this strategy may have been more advantageous in the ancestral past, given that we cannot rule out that FEPC may sometimes result in fertilization, it may remain advantageous to this day. In any case, if the benefit of FEPC is low yet costs are high, this raises questions as to why the behaviour persists. One explanation for why FEPC persists is that it is selectively neutral, that is, the benefits are low but the costs are also low. Given the costs outlined above, we suggest that this is unlikely. However, if costs are high, it is difficult to understand how a high-cost, low-benefit behaviour can persist. It is clear that the field would benefit immensely from explicit quantification of putative costs as well as potential reproductive benefits.

We did not find strong support for any of the other potential explanations. The CODE hypothesis suggests that FEPC is adopted by males to create an ‘environment of fear’ for females, who evolve the counter-strategy of pairing with a male partner for the protection and in return, offer access to copulation and fertilization [17]. While we found support for the prediction that males direct FEPC attempts towards unguarded females [17], presumably because of putative costs of escalated fighting with resident males, one could argue that targeting unguarded females would be expected regardless of the ultimate cause of FEPC. We did not find support for two other predictions of the CODE hypothesis, namely that FEPC is unrelated to a female’s fertility state and that female vulnerability to male aggression predicts where FEPC is focused [17]. On balance, we suggest that the CODE hypothesis probably does not explain FEPC in jackdaws. Additionally, we note that any male who does not engage in costly FEPC could still benefit from the social fear without paying the price. Thus, it is not clear how FEPC maintenance through CODE would be stable and not prone to invasion by cheats. We also found no strong evidence that males engage in FEPC when their own partner is fertile (in order to fertilize their partner with high-quality sperm), a key prediction of sperm turnover hypothesis. However, this is extremely tentative given the small sample sizes: three of five males engaged in FEPC when their partner was post-fertile, and two did so when their partner was fertile. Although our data do not allow us to make firm conclusions, we suggest that the sperm turnover is an unlikely explanation for FEPCs because other mechanisms (e.g. masturbation; Baker & Bellis [33]) could provide less costly means of ensuring ejaculate quality. Finally, key predictions of the byproduct hypothesis (FEPC as a pleiotropic behaviour) and the territory signalling hypothesis (males gain territory as a result of FEPC) could not be tested (see electronic supplementary material, table S1). Further work is also necessary to investigate these ideas.

We found ambiguous support for the reproductive suppression hypothesis, which suggests that males should target neighbouring females to decrease the reproductive success of direct competitors [30]. Reproductive suppression has previously been demonstrated in other birds. For example, in superb lyrebirds (*Menura novaehollandie* [69]) and white-winged choughs (*Corcorax melanorhamphos;* [70]), individuals destroy rival nests to suppress the reproductive success of direct competitors. Conspecific egg destruction may also occur in acorn woodpeckers (*Melanerpes formicivorus*) and Mexican jays (*Aphelocoma ltramarine*) [71,72]. In this context, the reproductive suppression hypothesis predicts that (i) females incur costs as a result of FEPC, (ii) this negatively influences their fitness outcomes and (iii) FEPC is undertaken by neighbouring males. Females may incur an injury during conflict with males [30], and given that FEPC occurs both during egg-laying and incubation (our populations [30]), eggs may also be damaged [30]. We could not quantify the costs of injury to females, but we did find a potential time cost of FEPC attempts. Specifically, females increased vigilance prior to the FEPC attempt, probably because they could hear or see an intruding male in close vicinity to the nest-box and decreased incubation. Interrupted incubation may lead to a sub-optimal adjustment of hatching synchrony to environmental conditions, which increases the likelihood of brood failure [73–75]. Considering that females are likely to experience more than one FEPC attempt per breeding season [30], this cumulative effect of short-term incubation disruption could potentially reduce fitness. However, since we only filmed each nest-box for a few hours and did not know the distribution of FEPC attempts across the breeding season, we could not test whether fitness was impacted by FEPC attempts. This should be the subject of future work. Additionally, our data indicated that FEPC attempts are undertaken by neighbouring males. Of the FEPC males we could identify, four of five were paired, breeding neighbours. Although this is consistent with the reproductive suppression hypothesis, it must be noted that males may be more likely to engage in extra-pair behaviour with their neighbours for a number of other reasons. For instance, males may be better able to judge the fertility state of neighbouring females by observing them. They may also be able to monitor the resident male’s activities more easily, thus decreasing their probability of being attacked during the FEPC attempt. Moreover, these putative advantages of mating with neighbours are not necessarily unique to FEPC but may also be advantageous where EPC is unforced. Indeed, in a species where EPC results in fertilization, chicks tend to be sired by neighbouring males [76]. Two further predictions of the reproductive suppression hypothesis are that FEPC is independent of the female’s fertile period and that male mate-guarding is not associated with female fertility. However, neither of these predictions were supported (our data [30]). In sum, this hypothesis deserves further investigation, but it would currently be premature to conclude that it is occurring in jackdaws. Potential limitations of the hypothesis are that (i) similar to the CODE hypothesis, it is open to cheats, as neighbouring males who do not engage in the behaviour could benefit from the reproductive suppression, and (ii) it is unclear why males would not simply destroy the eggs or kill the chicks of their neighbours.

It is also possible that FEPC can sometimes result in fertilization under specific ecological conditions. That is, benefits and costs (to males and females) vary spatially and/or temporally. For example, in resource-poor years, resident males may mate-guard less owing to increased foraging effort [30], while females may not have the energy to resist intruding males and simply accept copulations as a means of cost minimization [22]. Consistent with this, despite generally low rates of extra-pair paternity in jackdaws, rates do vary between populations: Gill *et al*. [30] found three potential but ambiguous cases of extra-pair paternity (which could also have been extra-pair maternity or egg-dumping) across 65 offspring; Henderson *et al*. [36] found no evidence of extra-pair paternity across 74 offspring; Liebers & Peters [37] found one likely case of extra-pair paternity across 39 offspring; Turjeman *et al*. [39] found 1 (potentially two) of 39 offspring were likely to be the product of extra-pair paternity or maternity, one ambiguous case of extra-pair paternity, maternity or egg-dumping and one brood with fully inconclusive results.

FEPC in species lacking penises has generally not been well studied, possibly because it is considered rare [23,77]. However, we found reports of FEPC in 48 bird species from 11 orders (of the 22 orders where species do not have penises). This is likely to be vastly under-representative because, in many species, copulation does not occur in the open and is therefore difficult to observe [22,78], and our criterion for inclusion in the FEPC database was fairly conservative. In any case, FEPC in species lacking penises can be considered taxonomically widespread. Despite a general lack of fine-scale quantification of FEPC behaviour across species, we were able to elucidate some broad patterns. Most species where FEPC has been observed are socially monogamous, and the majority are always or sometimes colonial breeders. This is consistent with previous work [17]. However, there is a caveat: most studies on extra-pair mating are conducted on colonially breeding species, and most colonially breeding species mate in the open, so FEPC is likely to be relatively obvious. Within colonial species, FEPC is frequently initiated by neighbouring males. However, whether this pattern is owing to specific targeting of neighbours, or just proximity and ease of access, is not clear. While it might be expected that FEPC would be more common in species where males are larger and can more easily overpower females with lower male injury risk, many species with FEPC show a little sexual size dimorphism. Indeed, it stands to reason that females would incur a higher cost of resistance if males were much larger and thus may instead accept EPC in order to minimize costs. Furthermore, data on fighting more generally shows that size-matched opponents tend to be more likely to escalate conflict [79]. In just under half the species identified, unforced extra-pair copulation was also recorded. This is also likely to be an underestimate, given that unforced extra-pair copulations are probably less obvious to observers, so may be more easily missed than FEPC. In species with unforced EPC, rejected EPC attempts could lead to FEPC. Nevertheless, based on current knowledge, it seems that FEPC does also occur in a range of species where unforced EPC does not occur (see electronic supplementary material Data).

While no studies have directly investigated whether FEPC increases siring success in species without a penis, data from western gulls (*Larus occidentalis*) [66], black-legged kittiwakes (*Rissa tridactyla*) [67] and jackdaws (here, [30]) suggests that FEPC rarely results in paternity. However, rates of FEPC do correlate with the rate of extra-pair paternity in the stitchbird [12,64]. Despite lacking a penis, the stitchbird is unusual in that the male cloaca rotates and swells to 400% of its original size during the breeding season, and a unique face-to-face copulatory position is adopted during FEPC [80]. Together, these behavioural and physiological adaptations are thought to enhance male stitchbird fertilization success during FEPC [12,80]. There is also some evidence to suggest FEPC increases siring success in American crows, where the rate of observed FEPC correlates with the rate of extra-pair paternity [65]. However, this is uncertain: the authors note that unobserved unforced EPC may have led to extra-pair fertilization instead [81]. The fact that FEPC may result in fertilization in some species lacking penises, while it appears not to in others, again highlights the need for in-depth study of this behaviour.

In conclusion, we found that the rate of extra-pair paternity is between 0 and 4% in our populations of jackdaws. Given that unforced EPC has never been observed in jackdaws, despite multiple detailed behavioural studies of wild populations, this indicates that 0–4% of chicks were sired through FEPC. This is in-line with findings from a previous study on a different population, where 87.5% of observed females experienced FEPC attempts in a single year, yet there was no (or potentially a very low level of) extra-pair paternity [30]. We tentatively suggest that either the immediate fertilization enhancement hypothesis or the relic hypothesis are the best supported explanations for why FEPC persists in jackdaws. However, acceptance of the relic hypothesis must be tempered by the putative costs of FEPC, as it is not clear how even small costs can be accompanied by no benefits. In order to better understand how FEPC persists, the costs of engaging in this behaviour must be quantified. We also find that FEPC is widespread across birds lacking penises; however, very little is known about FEPC in most species, and almost nothing about why it has evolved. Understanding the ultimate function of FEPC is, therefore, an important component of wider research into sexual behaviour. We suggest that the framework presented here, where specific predictions of multiple hypotheses are tested using both genetic and behavioural data, will help us understand the ultimate underpinnings of this widespread behaviour.

## Data Availability

Data and associated scripts can be accessed on Dryad [82]. Electronic supplementary material is available online [83].
